# Clinical Characteristics of 76 Patients with IgG4-Related Hypophysitis: A Systematic Literature Review

**DOI:** 10.1155/2019/5382640

**Published:** 2019-12-18

**Authors:** Yujuan Li, Hua Gao, Zhen Li, Xinxin Zhang, Yizhi Ding, Fengao Li

**Affiliations:** Department of Endocrinology and Metabolism, Tianjin Medical University General Hospital, Tianjin, China

## Abstract

**Background:**

IgG4-related hypophysitis (IgG4-RH) is a rare disease, and its prevalence remains unclear. In recent years, an increasing number of cases have been reported because of the increasing recognition of this disease. We aimed to summarize case reports of IgG4-RH and outline the clinical features and outcomes.

**Methods:**

We performed PubMed search of articles using the search terms “hypophysitis [AND] IgG4.” Consequently, only 54 English articles (76 cases) met Leporati's diagnostic criteria.

**Results:**

Of the 76 cases, the ratio of men to women was 1.5 : 1, and the age at diagnosis was 54.1 ± 17.8 years. The median IgG4 concentration was 405.0 mg/dl. Anterior hypopituitarism, isolated central diabetes insipidus, and panhypopituitarism were observed in 14 (18.4%), 12 (15.8%), and 44 (57.9%) cases, respectively. The sequence of anterior hormone deficiency was as follows: gonadotropin (68.4%), ACTH (63.2%), TSH (59.2%), GH (48.7%), and prolactin (42.1%). The median number of involved organs was 1.5, and the lung (18.4%), retroperitoneum (17.1%), kidney (15.8%), submandibular glands (14.5%), and pancreas (13.2%) were the common involved organs. Elevated IgG4 concentration and normal IgG4 level were in 42 (76.4%) and 13 (23.6%) cases, respectively. Patients with elevated serum IgG4 concentration were older (60.9 ± 14.3 vs 45.6 ± 17.4, *p*=0.001) and male-prone (78.6% vs 40.4%, *p*=0.003) and had a susceptibility of multiple organ involvement (78.6% vs 35.0%, *p*=0.001) compared to those with normal serum IgG4 levels. Males were older at disease onset (61.5 ± 12.6 vs 42.9 ± 18.8, *p* < 0.001) and had a higher IgG4 concentration (425.0 vs 152.5, *p*=0.029) and a greater number of involved organs (2.0 vs 0.0, *p*=0.001), while isolated hypophysitis was more prominent in female (63.3% vs 26.1%, *p*=0.001).

**Conclusion:**

In this review, we found that there were different characteristics between different genders. Patients with elevated serum IgG4 level in terms of some clinical features were also different from those with normal serum IgG4 level. However, the data in this review were limited by bias and confounding. Further clinical studies with larger sample sizes are warranted.

## 1. Introduction

IgG4-related disease (IgG4-RD) is a rare, newly recognized, multiorgan involved disease, which was characterized by infiltration of IgG4-positive plasma cells into the organs and elevated serum IgG4 level. The incidence of IgG4-RD was estimated to be 0.28–1.08/100,000 patients in Japan [1]. It was first created by Hamano et al. in 2001 when describing one case of sclerosing pancreatitis [2]. To our knowledge, pancreas, retroperitoneum, and salivary glands were the most commonly involved organs in IgG4-RD. Pituitary, which was a rare involved organ, was initially reported in 2004 [3]. It accounted for merely 1.5% of systemic cases of IgG4-RD [4]. Wong et al. reported the first case with both clinical manifestation and histopathologic evidence in 2007 [5]. In order to avoid the complication of transsphenoidal surgery, new diagnostic criteria were proposed by Leporati et al. in 2011 [6]. Since then, cases continued to be reported. In this review, we summarized case reports of IgG4-RH reported in the English articles and provided a detailed analysis of the clinical features of the 76 cases, aiming to get a better understanding of this recently recognized and rare entity.

## 2. Methods

This systematic review of the literature was conducted based on Preferred Reporting Items for Systematic Reviews and Meta-Analysis (PRISMA) guidelines [7].

### 2.1. Literature Search and Data Extraction

**Figure 1:**
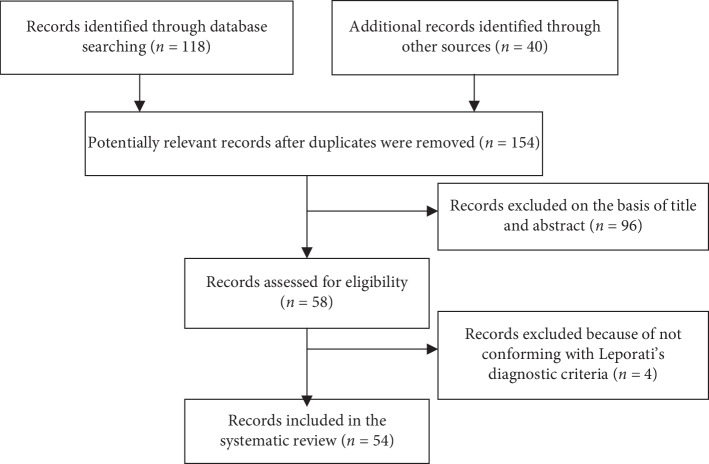
PRISMA flow diagram of literature search and selection.

We performed a PubMed database of the US National Library of Medicine search of articles using the search terms “hypophysitis [AND] IgG4” published up to March, 2019. A manual search of the literature was also performed. Articles were excluded if (1) cases were reported in languages other than English, (2) cases without individual data, and (3) cases not conformed with Leporati's diagnostic criteria (Table 1). Two investigators independently searched articles according to the inclusion and exclusion criteria. Any disagreements regarding the suitability of individual articles were resolved by discussion. Consequently, 54 articles of IgG4-RH (76 cases) were included. The PRISMA flow diagram is demonstrated in Figure 1. For each case, two investigators independently extracted the following parameters from eligible articles: sex, age, symptom, pituitary function, MRI, IgG4 serum concentration, involved organs, therapy, and the response to therapy. A list of all reviewed articles [3, 5, 6, 8–57] is given in Table 2.

### 2.2. Hormone Deficiency

Anterior hypopituitarism was defined as any anterior pituitary hormone deficiency. Secondary hypogonadism was diagnosed when FSH/LH concentration and estradiol or testosterone level was not elevated, respectively, for women or men. Secondary adrenal deficiency was defined as low morning serum cortisol and ACTH level. Secondary hypothyroidism was diagnosed when the serum FT4 level was below the normal range and serum TSH level was inappropriately low or normal. GH deficiency was defined as low age-adjusted IGF1 level. The diagnosis of diabetes insipidus was based on the clinical findings of polyuria and polydipsia, low levels of antidiuretic hormone (ADH), low urine osmolality in a water deprivation test, and an increase in urinary osmolality or a decrease in urine volumes in response to a desmopressin trial.

### 2.3. Statistical Analysis

All parameters were described in the standard summary statistics, including mean, standard deviation (SD), median, minimum, maximum, and composition ratio. Statistical differences for continuous, normally distributed data were analyzed by Student's *t*-test; all continuous, non-normally distributed data were analyzed by nonparametric testing (Mann–Whitney *U* test). Categorical variables were assessed by chi-square or Fisher's exact test, as appropriate. All statistical tests were performed by SPSS version 20. A *p* value <0.05 was considered statistically significant.

## 3. Results

### 3.1. Patient Demographics

**Figure 2:**
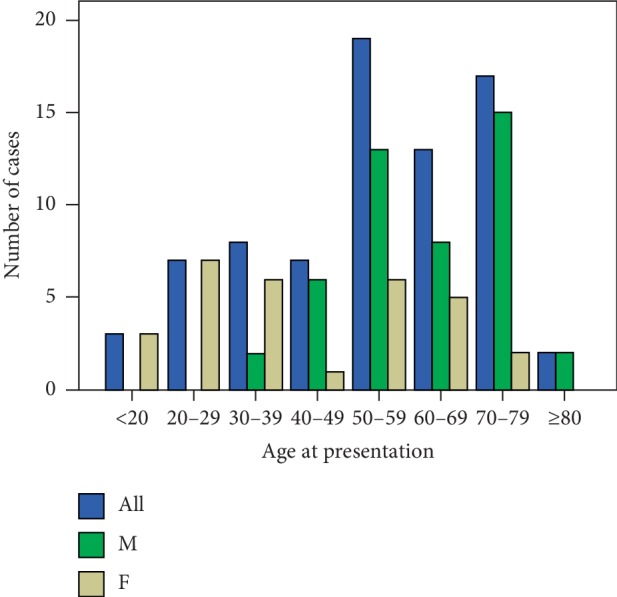
Age at presentation of IgG4-related hypophysitis.

In a total of 76 patients, the mean ± SD age at diagnosis was 54.1 ± 17.8 years. 60.5% (*n* = 46/76) were men (the mean ± SD age at time of onset was 61.5 ± 12.6 years), 39.5% (*n* = 30/76) were women (the mean ± SD age at time of onset was 42.9 ± 18.8 years), and the ratio of men to women was 1.5 : 1. The minimum age was 14 years (ranged from 14 years to 87 years). Two patients were pregnant women. According to the data, the ages of onset were as follows: 7 (9.2%) patients were in their 40s, 19 (25.0%) in their 50s, 13 (17.1%) in their 60s, and 17 (22.4%) in their 70s (Figure 2).

### 3.2. Main Symptoms

In summary, there were the following types of symptoms. Sellar mass effects (such as visual field loss and diplopia) accounted for 18.4%. Central diabetic insipidus (polyuria and polydipsia) accounted for 39.5%. Anterior hypopituitarism (decreased libido, amenorrhea, and bilateral galactorrhea) accounted for 10.5% and general symptoms (general malaise, headache, nausea, vomiting, fever, appetite loss, and weight loss) accounted for 40.8%. Certainly, headache (26.3%) was also caused by direct compression of pituitary mass.

### 3.3. Pituitary Function

Seven cases had no available data of anterior pituitary function, and 5 cases lacked description of posterior pituitary function in the 76 cases. Almost all cases of IgG4-RH showed central diabetes insipidus and/or hypopituitarism although three patients had no pituitary dysfunction. Various degrees of anterior hypopituitarism were observed in 14 (18.4%) cases. Isolated diabetes insipidus was diagnosed in 12 (15.8%) cases. Panhypopituitarism was recorded in 44 (57.9%) cases. Among patients with damaged anterior pituitary, gonadotropin was the most commonly deficient hormone (*n* = 52, 68.4%), followed by ACTH (*n* = 48, 63.2%), TSH (*n* = 45, 59.2%), GH (*n* = 37, 48.7%), and prolactin (*n* = 32, 42.1%). Of all cases, isolated ACTH deficiency (*n* = 1), isolated hypogonadism (*n* = 5), and isolated hypothyroidism (*n* = 1) were documented.

### 3.4. Serum IgG4 Concentration

In the 76 cases, 14 cases did not record the IgG4 level and 7 cases only showed normal without available data. Normal IgG4 level was defined as 140 mg/dl. Forty-two (76.4%) cases had elevated IgG4 concentration, when 13 (23.6%) cases showed normal IgG4 level (7 cases lack of detailed data). Among those patients who had available serum IgG4 level, the median IgG4 concentration was 405 mg/dl (*n* = 62). Comparing men with women, IgG4 concentration in men was higher (425.0 mg/dl vs 152.5 mg/dl, *p*=0.029).

### 3.5. Imaging Features

Analyzing pituitary MRI, 17 (22.4%) cases presented with pituitary mass. Twenty (26.3%) cases presented with thickened pituitary stalk. Thirty-nine (51.3%) cases showed pituitary mass and thickened pituitary simultaneously. In cases involving central diabetes insipidus, the bright signal seen in the posterior pituitary of T1-weighing imaging was absent.

### 3.6. Other Involved Organs

IgG4-RH is a part of IgG4-RD, and it commonly accompanied with other involved organs. From our data, 23 different organs or tissues were involved, with a median of 1.5 involved organs (range: 0–7). The lung was the most commonly involved organ (*n* = 14, 18.4%), followed by the retroperitoneum (*n* = 13, 17.1%), kidney (*n* = 12, 15.8%), submandibular glands (*n* = 11, 14.5%), and pancreas (*n* = 10, 13.2%) (Table 3). We observed that the number of an isolated pituitary lesion not associated with any systemic IgG4-RD was 31 (40.8%) cases.

### 3.7. Diagnostic Method

Forty-three (56.3%) cases were diagnosed by pituitary biopsy via a transcranial approach or transsphenoidal approach. Twenty-three (30.3%) cases were diagnosed based on biopsy-proven IgG4-related disease in other organs when enlarged pituitary gland and/or pituitary stalk were presented.

### 3.8. Therapy

There was only one not receiving treatment; moreover, there was little change in the follow-up of 4 years. The use of glucocorticoid alone was the most common therapy for our patients (*n* = 55, 72.4%). Various kinds and doses of glucocorticoid were used to treat this disease. However, there were still some cases showing a relapse of the pituitary mass when the doses of glucocorticoid were tapered (Table 4). Surgery combined with glucocorticoid was taken in 13 (17.1%) patients. Four (5.3%) patients undergone glucocorticoid combined with immunosuppressive or anti-CD20 agents, including methotrexate, mycophenolate mofetil, azathioprine, cyclosporine A, and rituximab. In cases where clinical outcomes were reported, 93.8% (*n* = 60/64) of cases showed improvement following treatment, at least initially.

### 3.9. Clinical Differences according to Sex

Compared with women, men were older at the age of onset and had a higher IgG4 concentration and a greater number of involved organs (*p* < 0.001, *p*=0.029, *p*=0.001). Another important difference between men and women was that the isolated IgG4-RH was almost exclusively present in women (*p*=0.001). However, they had no difference in efficiency of therapy (Table 5).

### 3.10. Clinical Differences according to Serum IgG4 Concentration

We analyzed the clinical features of patients according to elevated versus normal serum IgG4 concentration (Table 6). It showed patients with elevated serum IgG4 concentration were older, male-prone, and more inclined to multiple organ involvement compared to those with normal serum IgG4 levels (*p* < 0.01 for all comparisons).

## 4. Discussion

This review presented the largest number of IgG4-RH cases ever enumerated in English, with 76 cases meeting the inclusion criteria. We analyzed the characteristics of each parameter. In addition, we compared the differences of each parameter based on sex and serum IgG4 level aiming to understand the IgG4-RH precisely.

Our analysis showed IgG4-RH presented at the 6th decade of life (mean age 54.1 ± 17.8 years) and was associated with a 1.5 : 1 male predominance, by contrast with the common lymphocytic hypophysitis, which was common in young females, particularly in association with late pregnancy or the postpartum period, and peaked in incidence in the 4th decade of life [59]. Iseda et al. reported that the age of onset was 66.3 ± 9.8 years, and the ratio of men to women was 9.3 : 1 in 2014 [23]. Shikuma et al. reported the age of onset was 64.2 ± 13.9 years, and the ratio of men to women was 2.4 : 1 in 2017 [60]. The possible reason was one previously been diagnosed as primary hypophysitis met the histologic criteria of isolated IgG4-RH. Moreover, we found patients with isolated pituitary lesion more tend to be female (*p*=0.001). Importantly, 9 cases had been diagnosed IgG4-RD prior to IgG4-RH. We learned that it is important to follow-up these cases by considering them potential IgG4-RH cases.

With regard to features of MRI and pituitary function, the data characteristics were similar to Shikuma's review [60]. The sequence of anterior hormone deficiency to IgG4-RH was as follows: gonadotropin, ACTH, TSH, GH, and prolactin, which was different from lymphocytic hypophysitis that was characterized by ACTH > TSH > gonadotropin > prolactin > GH [61]. Elevated IgG4 concentration, as a common laboratory finding of IgG4-RH, was observed in 42 (76.4%) cases, and patients with elevated serum IgG4 concentration were older, male-prone, and more inclined to multiple organ involvement compared to those with the normal serum IgG4 level. This conclusion was consistent with the reports of Wallace and Carruthers [62, 63]. However, the subjects in their articles were IgG4-RD patients, which was different from our review. Certainly, the serum IgG4 level could elevate in noninflammatory conditions, such as Wegener granulomatosis, multicentric Castleman's disease, and idiopathic plasmacytic lymphoadenopathy [64] and could be normal in up to 40% of patients, who were diagnosed by biopsy-proven IgG4-related disease [65] and in postpartum IgG4-RH [48]. Moreover, low-dose steroid therapy may mask the evidence of systemic increases in the IgG4 level. Wallace et al. found there was no significant gender differences in IgG4-RD patients with regard to age at disease onset, disease severity, organ involvement, or serum IgG4 concentration [62]. Differently, we found males in IgG4-RH were older in age at disease onset, and male had a higher IgG4 concentration and a greater number of involved organs compared to female. The lung, retroperitoneum, kidney, submandibular glands, and pancreas were prevalent involved organs of IgG4-RH. We need to systematically explore these organs in IgG4-RH, especially patients with clinical symptoms of these common involved organs. Certainly, when patients had manifestations of other rare involved organs, we also need to explore them. IgG4-RH as a part of IgG4-RD, we also need to make a comprehensive exploration of the pituitary gland by assessing the level of pituitary hormone and doing a pituitary MRI examination when a patient was diagnosed with IgG4-RD.

Despite pituitary biopsy being an invasive examination and hard to operate, 43 (56.6%) cases were diagnosed by this method. We may reduce the necessity of pituitary biopsy by finding suspicious other organ damage with a careful examination. Currently, there was no clear standard for the treatment of IgG4-RH. Steroid therapy was the first-line treatment. The initial dose of prednisone was usually 0.6 mg/kg/day, and the dose could be regulated for rapid progression or higher body weight. It continued for 1-2 months. The dose was tapered to a maintenance dose (2.5–5 mg/day) over a period of 2-3 months, with a taper of 5 mg every 1-2 weeks. If disease relapsed, the physician might consider a maintenance dose for an extended period of time, up to three years, or a combination of immunosuppressants [66, 67]. For asymptomatic hypophysitis, we could regularly follow-up to evaluate the pituitary hormone level and pituitary MRI morphology. Patients with a lower pituitary hormone level or a larger pituitary gland than before or other involved organ may require cortical therapy. According to our data, 93.8% (*n* = 60/64) cases showed shrinkage of pituitary size after treatment. However, 5 cases of IgG4-RH relapsed when glucocorticoid were tapered at a low dose in all patients. All of them were men aged from 43 to 75 years. Tabata et al. [68] detected the changes of the serum IgG4 level in 44 cases of IgG4-RD and proposed that the serum IgG4 level can be a predictor of the relapse. We need to confirm it with more data in future.

While the exact etiology of IgG4-RH still remains unclear, its potential association with autoimmune conditions has been frequently reported. This review showed 5 cases (6.1%) were associated with autoimmune diseases, such as hashimoto's thyroiditis [25, 54], systemic lupus erythematosus (SLE) [17], and Sjögren syndrome [14]. It was reported that IgG4-RH could be misdiagnosed as malignancy, granulomatous diseases, or tuberculosis [69]. Moreover, the risk of cancer increased threefold in patients with IgG4-RD in comparison to the general population [70], and we should attach importance to this disease.

This review had certain limitations. On the one hand, this review was grounded on case reports and small case series, but case reports not published in the English language were excluded. In addition, some case reports described the clinical data and outcomes very briefly. Consequently, some of the variables were missing. These biases may have an influence on the conclusions of this review. On the other hand, reporting bias due to overreporting of severe cases may lead to an overestimation of the clinical disease. This bias could not be thoroughly ruled out. For rare diseases, such as IgG4-RH, we can establish an online disease registry system to facilitate systematic data collection furthermore.

## 5. Conclusions

We described clinical features of IgG4-RH. In addition, we revealed that there were different characteristics between different serum IgG4 levels and sexes. However, further clinical studies with larger sample sizes are warranted due to bias and confounding in this review.

## Figures and Tables

**Table 1:** Diagnostic criteria for IgG4-related hypophysitis.

1. Histopathology mononuclear infiltration of the pituitary gland, rich in lymphocytes and plasma cells, with more than 10 IgG4-positive cells per high-power field
2. Sellar mass and/or thickened pituitary stalk on pituitary MRI
3. Biopsy-proven involvement in other organs (association with IgG4-positive lesions in other organs)
4. Elevated serum IgG4 levels (>140 mg/dl)
5. Rapidly reduction of the pituitary mass and symptom improvement with steroids
When any of the following is fulfilled, criterion 1 only, criteria 2 + 3, or criteria 2 + 4 + 5.

**Table 2:** Summary of the reported cases of IgG4-related hypophysitis.

Case no	Age	Sex	IgG4 (mg/dl)	Pituitary function		MRI		Number of other affected organs	Biopsy		Therapy	Response		Authors
				AH	DI	Stalk	Pituitary		Pituitary	Others		Symptom	MRI	
1	66	F	485	+	−	−	+	4	−	—	GC	+	+	van der Vliet [3]
2	71	M	405	+	+	−	+	3	−	Submandibular gland, retroperitoneum	GC	+	+	Tanabe et al. [8]
3	70	M	2220	+	−	+	−	2	−	Submandibular gland	GC	+	+	Yamamoto et al. [9]
4	77	M	720	+	−	−	+	2	+	Pancreas, cholecyst	Surgery + GC	nd	nd	Wong et al. [5]
5	55	M	1860	+	+	+	−	2	−	Paranasal sinus	GC	+	+	Isaka et al. [10]
6	62	M	292	+	+	+	−	3	−	Lung	GC	+	+	Tsuboi et al. [11]
7	74	F	nd	+	+	+	+	1	+	—	Surgery + GC	+	+	Osawa et al. [12]
8	70	M	924	+	+	+	−	3	−	Liver, lung, parotid gland	GC	+	+	Hori et al. [14]
9	68	M	159	nd	+	+	+	2	−	—	GC	+	+	Haraguchi et al. [13]
10	74	F	170	+	+	+	+	1	−	Lymph node	GC	+	+	Haraguchi et al. [13]
11	55	M	127^*∗*^	+	+	+	+	3	−	Lacrimal gland, kidney	nd	nd	nd	Patel and Szostek [16]
12	71	M	240	−	+	+	+	1	−	Pleural	GC	nd	nd	Nagai et al. [15]
13	75	M	nd	+	+	+	+	1	+	Paranasal sinus	GC	+	+	Leporati et al. [6]
14	47	M	94.9	nd	nd	−	+	3	+	Lung, lymph node	GC	+	+	Nishina et al. [17]
15	53	F	Normal	+	−	−	+	0	+	—	Surgery + GC	+	+	Kanoke et al. [20]
16	60	F	Normal	+	−	+	+	0	+	—	Surgery + GC	+	+	Kanoke et al. [20]
17	66	M	nd	+	−	+	−	4	+	Mediastinum	Surgery + GC	+	+	Hsing et al. [19]
18	55	M	1010	−	−	+	+	0	+	—	GC	+	+	Hattori et al. [18]
19	53	M	24.5	+	+	+	−	3	−	Lung	GC	nd	+	Bando et al. [21]
20	76	M	513	+	+	+	+	0	−	—	GC	nd	+	Bando et al. [21]
21	58	F	405	nd	+	+	+	3	−	Lung, stomach	GC	nd	+	Bando et al. [21]
22	68	F	16.9	+	−	+	−	0	+	—	GC	nd	+	Bando et al. [21]
23	53	F	16.9	+	+	+	+	1	+	—	GC	nd	+	Bando et al. [21]
24	67	F	82.5	+	+	+	+	0	+	—	GC	nd	+	Bando et al. [21]
25	76	M	2100	+	+	+	+	2	+	Pancreas	GC	+	+	Iseda et al. [23]
26	33	F	nd	+	+	+	−	0	+	—	Surgery + GC	+	+	Khong et al. [24]
27	70	M	300	+	+	+	+	3	+	Retroperitoneum	GC	+	+	Ohkubo et al. [25]
28	40	M	413	+	+	+	+	1	+	Lacrimal gland	GC + azathioprine	+	+	Caputo et al. [22]
29	37	F	nd	+	+	+	+	0	+	—	GC	+	+	Sosa et al. [26]
30	25	F	nd	+	−	+	+	0	+	—	GC	+	+	Sosa et al. [26]
31	71	M	nd	−	+	+	+	0	+	—	nd	nd	nd	Imber et al. [28]
32	87	M	285	+	+	+	+	0	−	—	GC	+	+	Nakasone et al. [30]
33	32	F	nd	+	+	−	+	0	+	—	GC			Tauziede-Espariat et al. [32]
34	38	F	Normal	+	+	−	+	0	+	—	GC	+	+	Tauziede-Espariat et al. [32]
35	43	M	79.2	+	−	+	+	1	+	—	GC	+	−	Ngaosuwan et al. [31]
36	67	M	240	+	+	+	−	2	−	Liver	GC + azathioprine	+	+	Joshi et al. [29]
37	62	M	307	−	+	+	+	2	−	Kidney	GC	nd	nd	Joshi et al. [29]
38	72	M	853	+	+	+	−	1	−	Kidney	GC	+	+	Harano et al. [27]
39	38	F	377	+	+	−	+	2	+	Orbita	GC	+	+	Alexandraki et al. [33]
40	55	M	800	+	+	+	+	0	+	—	GC	+	+	Bhagwat et al. [34]
41	16	F	nd	−	+	+	+	0	+	—	GC	nd	nd	Decker et al. [35]
42	54	M	nd	+	+	+	+	2	−	Kidney	GC	+	+	Patel et al. [36]
43	76	M	1030	+	−	+	+	0	+	—	GC	+	+	Anno et al. [37]
44	57	M	83.9	+	+	+	+	5	+	—	GC + azathioprine	+	nd	Gu et al. [38]
45	29	F	135	+	+	−	+	0	+	—	Surgery + GC + MMF	+	+	Hadjigeorgiou et al. [39]
46	56	M	570.7	+	+	+	+	2	−	—	Surgery + GC	+	+	Huang et al. [40]
47	70	M	425	+	+	+	−	4	−	Lip	No	—	—	Kawasaki et al. [41]
48	51	F	nd	+	+	+	+	0	+	—	Surgery + GC	+	+	Lee et al. [42]
49	85	M	713	nd	+	+	−	1	−	Kidney	GC	+	+	Matsuda et al. [43]
50	36	F	nd	+	+	+	+	0	+	—	Surgery + GC	+	nd	Rotondo et al. [44]
51	49	M	nd	+	−	+	+	0	+	—	GC	+	+	Rotondo et al. [44]
52	56	M	298	+	−	+	+	3	−	—	GC	+	+	Yatabe et al. [45]
53	14	F	61	−	−	+	+	0	+	—	GC + rituximab	−	+	Bullock et al. [46]
54	25	F	55.7	+	+	+	+	0	+	—	GC	+	+	Koide et al. [48]
55	23	F	nd	+	+	−	+	1	+	Retroperitoneum	Surgery + GC	nd	nd	Guarda et al. [47]
56	50	M	426	+	+	−	+	7	+	—	GC	−	−	Liu et al. [49]
57	16	F	43.7	+	+	+	−	2	−	Submandibular gland	GC	−	−	Liu et al. [49]
58	57	F	2250	−	+	+	−	3	−	Lacrimal gland	GC	+	+	Liu et al. [49]
59	36	M	2470	−	+	+	−	4	−	Lacrimal gland	GC	+	+	Liu et al. [49]
60	44	M	1910	−	+	+	−	3	−	Pancreas	GC	+	+	Liu et al. [49]
61	46	M	327	+	+	+	−	1	−	Lymph node	GC	+	+	Liu et al. [49]
62	51	M	1980	−	+	+	−	3	−	Submandibular gland	GC	+	+	Liu et al. [49]
63	58	M	5410	+	+	+	−	4	−	—	GC	+	+	Liu et al. [49]
64	64	M	199	+	+	+	+	0	−	—	GC	+	+	Liu et al. [49]
65	61	M	4680	+	+	+	+	2	−	Paranasal sinus	GC	+	+	Liu et al. [49]
66	58	F	58	+	+	+	+	0	+	—	GC	+	+	Murphy et al. [50]
67	36	M	Normal	+	+	+	+	0	+	—	GC	+	+	Sosa et al. [51]
68	67	F	865	nd	+	+	−	6	+	Bladder	GC	+	+	Xue et al. [53]
69	50	M	13.1	nd	nd	−	+	0	+	—	Surgery	+	+	Tang et al. [52]
70	24	F	Normal	+	+	+	+	0	+	—	Surgery + GC	+	+	Yuen et al. [54]
71	24	F	Normal	nd	nd	−	+	0	+	—	GC	+	nd	Yuen et al. [54]
72	71	M	Normal	+	+	−	+	0	+	—	GC	+	−	Yuen et al. [54]
73	78	M	802	+	nd	−	+	4	−	—	Surgery + GC	+	+	Kanie et al. [56]
74	43	F	462	+	+	+	+	2	−	Orbita	GC	+	+	Yoshida et al. [58]
75	28	F	880	−	−	−	+	0	−	—	Methotrexate + rituximab	+	nd	Goulam-Houssein et al. [55]
76	65	M	221	+	nd	−	+	0	+	—	Surgery + GC + rituximab	+	+	Vauchot et al. [57]

F, female; M, male; nd, not described; AH, anterior hypophysitis; DI, diabetes insipidus; GC, glucocorticoid; MMF, mycophenolate mofetil.

**Table 3:** Other involved organs.

Organ involvement	Number of cases	Frequency (%)
Lung	14	18.4
Retroperitoneum	13	17.1
Kidney	12	15.8
Submandibular glands	11	14.5
Pancreas	10	13.2
Lacrimal glands	10	13.2
Parotid glands	8	10.5
Lymph nodes	8	10.5
Paranasal sinus	4	5.3
Orbita	3	4.0
Cholecyst	3	4.0
Thyroid	2	2.6
Salivary glands	2	2.6
Liver	2	2.6
Bladder	2	2.6
Eyes	2	2.6
Pachymeninx	2	2.6
Pericardium	1	1.3
Lip	1	1.3
Intestine	1	1.3
Mediastinum	1	1.3
Paraspinal muscle	1	1.3
Pleura	1	1.3

**Table 4:** Characteristics of recurrent cases.

No.	Sex	Age	Relapse-free interval	Dose of relapse	Postrelapse treatment	References
1	M	75	3 months	Below 10 mg/d	Prednisolone 15 mg/d	[6]
2	M	70	8 years	5 mg/d	Prednisolone 30 mg/d	[25]
3	M	43	12 months	7.5 mg/d	Not described	[31]
4	M	57	4 months	Not described	Rituximab	[38]
5	M	56	7 months	Below 7.5 mg/d	Prednisolone 40 mg/d	[45]

**Table 5:** Characteristics of patients with IgG4-related hypophysitis according to sex.

	Male	Female	*p*
Age at diagnosis, mean ± SD (range) years	61.5 ± 12.6 (36–87)	42.9 ± 18.8 (14–74)	<0.001
Median IgG4 concentration (mg/dl)	425.0	152.5	0.029
Median number of involved organs	2.0	0.0	0.001
Isolated hypophysitis	26.1%	63.3%	0.001
Efficiency of therapy	92.3%	96.0%	1.0

**Table 6:** Characteristics of patients with IgG4-related hypophysitis according to elevated versus normal serum IgG4 concentration.

	Normal	Elevated	*p*
Age at diagnosis, mean ± SD years	45.6 ± 17.4	60.9 ± 14.3	0.001
Sex, male	40.4%	78.6%	0.003
Multiorgan involvement	35.0%	78.6%	0.001
Isolated hypophysitis	65.0%	21.4%	0.001
Efficiency of therapy	82.4%	97.3%	0.165
